# Ubuntu: Developing Future STEM Leaders With Michigan's Leadership and Technology Global Course in Cape Town

**DOI:** 10.1002/yd.70047

**Published:** 2026-02-17

**Authors:** Natasha T. Turman

**Affiliations:** ^1^ Women in Science and Engineering Residence Program College of Literature, Science, & the Arts University of Michigan Ann Arbor USA

## Abstract

This article explores the conceptualization, design, and implementation of the University of Michigan's inaugural Leadership and Technology Global Course in Cape Town, South Africa. This 3‐week study abroad experience centered the perspectives of gender‐minoritized leaders in science, technology, engineering, and math (STEM) to demonstrate the importance of diversity, inclusion, and cultural competency for effective leadership practice. The values of *Ubuntu* (i.e., interconnectedness, community, compassion/empathy, responsibility, reconciliation/healing) frame the themes that emerged each week of the course and illuminate leadership best practices. With the voices of the 12 participants foregrounded, this article presents counternarratives, to disrupt both leadership and study abroad stories most often told.

## 1 Introduction

*Ubuntu* is a term, a philosophical framework from sub‐Saharan African Nguni Bantu language with historical roots in several South African cultures (Udah et al. 2025). Ubuntu is often translated to mean ‘I am because we are’ and reflects the interconnectedness of individuals and how one's humanity manifests in relationship to others (Tutu 2010). When considered alongside leadership practice and development, Ubuntu magnifies the relational, collective, and social change capacities one can harness to positively effect change. The twelve students who journeyed to Cape Town, South Africa from the University of Michigan for the Leadership and Technology Global Course Connection (GCC) program, learned firsthand, what Ubuntu looks like in practice and expanded their leadership capacities in more inclusive, equity‐minded, and culturally enriching ways.

This article provides an examination of the creation and execution of the inaugural Leadership and Technology GCC at the University of Michigan, which took place in the spring semester of 2025. The global experience showcased the intercultural learning and leadership development of undergraduate students pursuing an array of STEM and non‐STEM majors. The course's development, key curriculum, programmatic elements, and impactful student‐centered leadership lessons are discussed.

### 1.1 Author's Positionality: A Dream Deferred

I studied Chemistry in undergraduate at the incomparable Spelman College, whose mission is to educate, elevate, and center gender minoritized individuals across the African Diaspora. I imagined what studying abroad would look like but, I thought, like many STEM students do, that it would derail my course completion and was expensive (Witenstein and Hastings 2018). Most of all, if I am being honest, I did not see a critical mass of young Black and Brown faces like mine pursuing study abroad opportunities during college (Smith et al. 2013; Wanger et al. 2020).

The number of Black students from the United States (US) participating in study abroad experiences has ebbed and flowed over the years–3.5% in the early 2000s and rising as high as 6.4% in 2018–2019 (National Center for Education Statistics (NCES)). 2020–2021 NCES data revealed approximately 4% of Black US students studied abroad, only a 0.5% increase from the early 2000s; juxtaposed to White US students who comprised almost 70% of the total number of students studying abroad in 2020–2021(National Center for Education Statistics 2022). This significant gap highlights the need for more dialogue, institutional interventions, and support to increase the number of racially diverse students studying abroad from the US (Wanger et al. 2020).

It wasn't until my doctoral studies, several years later that I was able to experience studying abroad. This experience confirmed for me the importance of global learning. For the past 17 years as a higher education administrator, instructor, and scholar, I have strived to curate dynamic learning experiences. Since 2019, I have led the Women in Science and Engineering Residence Program (WISE RP) at the University of Michigan, where I support the identity and leadership development of emerging STEM professionals.

My research and practice are grounded in examining how leadership development manifests for college‐aged students. Through critical frameworks, students are exposed to the ways identity, context, power, privilege, and systems inform and shape their overall STEM experiences (Dugan 2017; Turman and Pierre 2025). Cultivating leaders who are critical thinkers, who can engage within and across differences, and who have the skillsets to engage in culturally competent and relevant work, can positively transform STEM fields (Turman and Pierre 2025). With this goal in mind, the University of Michigan's Leadership & Technology Global Course Connection (GCC) program came to fruition.

## 2 Program Creation: The Counternarrative

When I began designing this global learning experience, I was open to the site location. However, I was cognizant of the deficit in my own linguistic capital (Yosso 2005) and felt more efficacious as an instructor guiding students in a country that was receptive to English speaking learners. The University of Michigan's Center for Global and Intercultural Studies (CGIS) colleagues suggested three places they felt aligned with the course's goals and priorities: Denmark, Singapore, and Cape Town.

After surveying the existing GCCs at the University of Michigan I learned that all were in European, Asian, or South American countries. According to a recent US News and World Report article, 64% of students studying abroad from the US did so in European countries; particularly Spain and the United Kingdom (Wood 2025). Yet, in 2023–2024, only 1.2% of US study abroad students did so in South Africa (Wood 2025). This statistic is staggering given the expansiveness and diversity of the continent of Africa. Armed with this information, I sought to disrupt the dominant narrative of what study abroad looks like and where it takes place.

I was immediately drawn to Cape Town, South Africa. South Africa has a rich history, marred by the enslavement of Black and indigenous peoples, distinctively transformed by the political oppression and social unrest of the Apartheid, while in the present day, shaped by the racial, ethnic, and class hierarchy that persists in the country (Ross 2008). It is beautifully shaped by the legacies of influential leaders like Nelson Mandela, Desmond Tutu, Winnie Madikizela‐Mandela, and Lillian Ngoyi who all rallied for change and left a legacy on the country and world. Highlighting the rich history and legacy, while displaying the powerful and intersectional STEM work and leadership that manifested across the country of South Africa and in particular, Cape Town was the opportunity of a lifetime.

### 2.1 Leadership and Technology GCC Overview

Science, technology, engineering and math are often coupled together and simplified to ‘STEM’. However, each discipline has its own origins, nuances, and personality. When STEM is distilled down to its individual parts and explored in diverse environments and through varied perspectives, its uniqueness is revealed to enrich learning and deepen the field's overall contributions. Cape Town is considered the “Silicon Valley” of South Africa (Nwankwo 2022, para 3) and is ranked among the world's top 100 emerging start‐up ecosystems (Shoke 2024). The program's goal was to elevate this unique positionality to facilitate cultural competency and leadership development, exposing students to the ways technology globally shapes and informs facets of the public good.

This program specifically centered the perspectives of gender‐minoritized leaders in technology and engineering to examine how important diversity, inclusion, and cultural competence are for effective leadership practice. Students were given space to reflect on their own leadership identities and the social structures that affect those identities; while engaging with dynamic leaders to discover the strategic ways leaders leverage unique capital to develop leadership competencies and skills to affect change.

What sets a GCC apart from most study abroad experiences is the “CC” or the ‘Course Connection.’ The Leadership and Technology GCC and field course ALA 270 Leadership STEM seminar, were designed to connect to and expand the on‐campus academic curriculum of ALA 108: STEM Success. ALA 108 focuses on one of WISE RP's core tenets, cultivating critical leadership, with the primary goal of building students’ leadership capacities and competencies (e.g., communication, cultural competency, empathy, ethics, leadership, lifelong learning, systems thinking, and teamwork; Guthrie and Devies 2024). The GCC field course and seminar delved deeper to broaden themes around gender, identity politics, intersectionality, cultural competency, and leadership development in STEM. Each course prioritized students’ self‐exploration and equipped students with tools and language to find their voice as leaders broadly and within STEM environments.

## 3 Program Design and Implementation

The University of Michigan's GCC program's 21‐day structure appeals to an array of students, especially those in STEM. During the initial pitch of a STEM focused study abroad experience in 2023, I surveyed the various program types available in my college and decided on the GCC format. CGIS colleagues highlighted key benefits of the 21‐day GCC program structure. First, (1) Accessibility: it is as long and meaningful as possible while keeping costs down for students; (2) Time for global adjustment: students take at least a week to acclimate to a new international context, a 3‐week structure ensures they have adequate time to engage and immerse themselves in the environment; (3) Deeper than tourism: s allows students to move beyond surveying and touring, to become a part of the community; (4) Creates space for additional learning opportunities: the start and end of the GCC is limited to the month of May, allowing students a full summer to engage in other activities like classes, jobs, internships, or research; and, (5) Time to explore: the GCC model creates a healthy balance between required instructional contact hours and independent site exploration. Each of these benefits informed the site selection, curricular, and co‐curricular aspects of the Leadership and Technology GCC.

Twenty‐one days is a ‘sweet spot’ for students with limited time/bandwidth, like those in STEM who often forgo studying abroad because of course curriculum limitations, financial barriers, or perceived limited utility to their overall academic pursuits (Stearns 2009; Witenstein and Hastings 2018). The ability to immerse oneself in a new culture and geographic location while in community with other students who share similar interests and also navigate the complexities of STEM, creates shared context that can positively disrupt notions of isolation, anxiety, and fear to venture abroad.

### 3.1 If You Build It, They Will Come, Right?

This global experience was originally exclusively for gender‐minoritized students in the WISE RP. I assumed that having an ideal program structure and an already captive audience in an established academically anchored living and learning community would have made recruitment for this experience easy. However, the 2024 GCC did not occur due to insufficient enrollment. To assist with future planning, a survey was administered to the WISE RP community. It revealed that there were preconceived notions about Africa — (“too far,” “not safe,” “requires special medicine”). Narratives like these are often influenced by media, stereotypes, and implicit/explicit biases (Mayer 2025).

When I reapplied for 2025, I opened the GCC to the entire campus community. This shift sparked the curiosity of an array of students from all backgrounds, with diverse identities, lived experiences, and STEM pursuits. The program received 24 applications; 18 were admitted. A total of 12 students completed their commitment form by the deadline. Of the 12 students participating, 67% identified as Black or multi‐racial and two identified as male. Surprisingly, over half were not members of the WISE RP.

In partnership with the Institute for the International Education of Students (IES Abroad), a preeminent not‐for‐profit study abroad and internship organization, the logistical, cultural, and co‐curricular components of the program came to life. Twenty‐one days, six instructor lead lectures, five‐cultural excursions, three‐guest lecturers, three‐industry site visits, three‐written reflections, two‐community based learning experiences, one‐photo elicitation final project and a host of meals and self‐guided explorations, rendered a collection of memories, core learning, and critical leadership development. The next section discusses how the Leadership and Technology GCC promoted learning, increased cultural competency, heightened appreciation for diverse perspectives, and expanded leadership capacities for students.

## 4 Ubuntu and STEMLeadership Actualized

There are several interpretations and values that individuals have ascribed to the indigenous African philosophy of Ubuntu. For the purposes of this article, I will leverage the following values of Ubuntu (i.e., interconnectedness, community, compassion/empathy, responsibility, reconciliation/healing; Ngubane and Makua 2021; Udah et al. 2025). These chosen values provide a rich lens to understanding the land we traversed, the history we examined, and the leaders we learned from during our time in Cape Town. This article utilizes the critical framework of community cultural wealth (Yosso 2005), to ground the leadership, sociopolitical, and cultural phenomena observed in Cape Town. Yosso's (2005) acclaimed work suggests that Communities of Color harness cultural wealth by relying on diverse forms of capital (i.e., aspirational, navigational, social, linguistic, familial, and resistant) to thrive strategically and successfully.

The narratives constructed and findings offered are anchored by this critical framework to reveal the nuances of leading, doing STEM, and affecting change, from the margins. Drawn from students’ weekly written reflections, each section opens with a student's narrative to center their voice. Through Ubuntu's values, core curriculum and activities are highlighted to illuminate the unique approaches and innovations of STEM leaders in Cape Town.

### 4.1 Week One: Reconciliation and Healing

When I told some people that I was coming to Cape Town, they asked me, “Out of all of the countries that you could go to in Africa, why would you choose Cape Town, [South Africa]?” I never understood why people make assumptions about places they have never been to, but that day taught me that there is so much beauty in places that you would never expect. ∼ A.L.

Week one of the GCC was like a pilgrimage. In addition to the excitement of being in a new place, we quickly discovered that to fully immerse, we needed to first ground ourselves, detox from some personal ideals, and unlearn some viewpoints that may have influenced our perceptions of South Africa and its people. Ubuntu's values of reconciliation and healing suggest that in the presence of conflict/dissent, one must center restorative measures, dialogue, and social justice to heal and reconcile.

### 4.2 Unlearning the Single Story

We kicked off the GCC learning experience discussing identity, culture, and the dangers of a single story while enveloping ourselves in the beautiful history and sites of Cape Town. We listened attentively to a political science professor from the University of the Western Cape who gave us a snapshot into the history of South Africa, the social construction of race, and how the historical underpinnings shaped its past, present, and future. The insights shared prompted students to reflect on semester course conversations about intersectionality and the compounding effects of multiple marginalized identities. Like Yosso (2005) suggested in the community cultural wealth model, as new study abroad visitors, we needed to leverage our resistant capital to push against dominant norms and values and systemic ideals that could hinder our ability to successfully exist in this new space. One student thoughtfully captured this resistant capital in their week one reflection, sharing:

I was especially impacted by our discussions around Chimamanda Adichie's concept of “the danger of a single story.” Her words reminded me how often we reduce individuals, cultures, and communities to limited narratives usually shaped by media, assumptions, or lack of exposure. I began to think critically about the narratives I hold about others, as well as the ones projected onto me. One key lesson I took away is that leaders must intentionally disrupt stereotypes not only by amplifying diverse stories but also by being open to learning from experiences that challenge their assumptions. ∼N.O.

As we walked through the historic streets of Cape Town, we were reminded of the power of intersectionality and the “interconnectedness of people, social problems, and ideas” (Hill Collins 2019, p. 2). We discovered the women leaders behind key South African movements. These women did not receive the recognition deserved; in part because of dominant male leadership prototypes, but their leadership was invaluable in promoting change in South Africa. We stood in the center of Church Square, the historic open market site where enslaved people were sold and purchased, and where now eleven granite memorial blocks sit to honor those enslaved. Not only did we discover how the people of South Africa are still actively healing and reconciling their political, social, and cultural dissonance, but as visiting learners, we too found ourselves healing during the unlearning and learning processes.

### 4.3 Leading the Next Generation: Community‐Based Learning

Our first community‐based learning excursion supported the efforts of the Cape Town Science Center. Through engagment with younger STEM learners, GCC students were able to put into practice leadership competencies (e.g., teamwork, empathy, communication, lifelong learning) to discover firsthand how they are also responsible for shaping the future of STEM. Ubuntu teaches us that everyone has a role to play and is essential to the process. As this week concluded, we learned how our lives and histories are intricately linked to one another and that to grow ourselves and others, we must be open to the principles of Ubuntu.

### 4.4 Week Two: Community and Interconnectedness— A Shift From ‘I’ to ‘We’

This week in Cape Town has already challenged my thinking and expanded my understanding of social, environmental, and cultural issues in profound and unexpected ways. During the Future Water lecture, I was moved by the emphasis on the importance of community involvement, approval, and engagement when implementing any kind of development project—especially when those projects directly affect people's lives, access to resources, and relationship to land. ∼K.P.

Two core values of Ubuntu are community and interconnectedness (Udah et al. 2025); two sides of a proverbial coin. Community emphasizes collective well‐being over individualism (Tynan 2021). Interconnectedness stresses the relationship between people, the environment, and spirit, acknowledging that all actions shape the entire community (Seehawer 2023; Udah et al. 2025). Udah et al. (2025) articulated this dynamic when they posited, “the individual's existence, therefore, is predicated on the existence of the community, and likewise, the community thrives on the interconnectedness and contributions of its members” (p. 440). As we delved into week two of the GCC, we were able to directly experience these two core values.

### 4.5 STEM Leadership and the Collective Good: Future Water Initiative

Water is a constitutional right in South Africa! We were amazed to learn this during our visit to the Future Water Initiative at the University of Cape Town. We engaged with transdisciplinary researchers whose mission was to reimagine the future of water for all of South Africa. Future Water brought together brilliant minds across STEM and humanity disciplines alongside community thought partners whose indigenous knowledge systems provided a more critical and expansive account of the effects of and viable solutions for the water crisis in South Africa. We also learned of the powerful matriarchal influence rural women have on the land and communities.

This visit set the tone for the rest of the week, centering relationships, community, and demonstrating how STEM professionals and leaders inform the public good. An excerpt from a student's reflection captured this impact best saying,

I had never realized that engineering careers could contribute to society in such a humanitarian way, and I think pursuing a career that intertwines the two, such as the work done at Future Water, is definitely something I want to look into more. ∼C.O.

It was at this moment that C.O.’s aspirational capital grew, yielding an increased hope for what possibilities lie ahead, regardless of potential barriers/resistance (Yosso 2005).

### 4.6 Innovation Meets Opportunity: WomHub

One of the best catalysts for leadership and STEM efficacy building is through vicarious experiences (Rittmayer and Beier 2008). After our visit to Future Water, students engaged with engineering entrepreneurs who were merging their technical backgrounds, creativity, and duty for civic engagement to innovate. WomHub, a boutique incubator, accelerator, and co‐working space for female founders in STEM* (science, technology, engineering, and mining), left a lasting mark on students. Ten out of twelve reflections for the week highlighted key takeaways from WomHub and a desire to facilitate similar outcomes once back in the US.

After students engaged with female founders in STEM* who participated in WomHub's accelerator and incubator programs for entrepreneurs, one student shared this insightful reflection:

When it comes to the true essence of what STEM leadership is I feel I learned quite a lot from the innovator of the woman's sanitary menstrual products at WomHub. Through her work she is making amazing contributions to end the concurring era of period poverty to allow women to continue pursuing life to the fullest without the worry of bleeding in public or health risks with these products at hand. When learning her story, I was inspired by how she used her talents and passion to help others in her community through her career as a founder of a product. As I continue to study to establish a STEM career for myself in the future I have for a long time felt determined to similarly find a way to help my community through my foundation in STEM. ∼A.O.

### 4.7 ‘I’ to ‘We’: Soil for Life and Bo‐Kaap

Our second week ended with two touch points that embodied interconnectedness and community—of the land and of the people. As a visiting learner, it can be easy to fall into tourist mode if not deliberate. To prevent this, students in the GCC engaged in cross‐cultural leadership development through service learning. ‘Peace begins in me’ is the mantra posted overtop the entrance of Soil for Life, a not‐for‐profit organization whose mission is to empower individuals to cultivate the necessary skills and resources to provide for themselves and their communities. Students got their hands dirty, literally, and their hearts and minds opened to how they can bring about change in their communities. Soil for Life daily actualizes Ubuntu's value of interconnectedness, believing that “a healthy world rests on re‐establishing the harmony between the earth and its people” (About: Soil for life 2024, para 7).

Week two concluded with one of the most memorable touchpoints of the entire experience: The Bo‐Kaap tour and cooking experience. The Bo‐Kaap neighborhood in Cape Town is known for its Cape Malay history and culture. It was once known as Malay Quarter, a racially and religiously segregated area on the hills of downtown Cape Town. During Apartheid, it was an exclusive area for Muslims. Known for its bright colorfully painted houses, which symbolized the freedom of enslaved people once allowed to purchase homes in that area, Bo‐Kaap was indeed a welcome sight.

We were invited into the home of Master Chef Zainie Mesbach. Through this gracious invitation, students garnered a deeper appreciation for Bo‐Kaap culture and discovered the interconnectedness of Cape Malay people to the rest of South Africa and the world. Chef Mesbach leveraged her familial capital by integrating her adult children into the experience to teach the history of their peoples and lead us in a shared cooked meal. The lessons offered deepened our understanding of the systemic barriers Indigenous Cape Malay people, and their descendants, still face in Cape Town. Their narratives illustrated the power of harnessing resistant capital to ensure the values and culture remain pure to Bo‐Kaap. The experience was nothing short of inspiring.

Each experience in week two taught a unique and powerful lesson on leadership. Specifically, we learned the vital role community, interconnectedness, and empathy plays in leading to affect change. We saw the positive effects of counternarratives, and the benefits gained when students are immersed in a culture. As one student reflected,

I learned the value of storytelling in leadership this week, particularly in historically significant neighborhoods like ‘Bo‐Kaap.’ I realized that every community has a unique tale to tell. These tales foster empathy and can help leaders make more deliberate, people‐focused choices…Listening to people's stories, values, and life experiences is the first step in genuinely understanding them as a leader. ∼J.U.

With an eye toward the third and final week of the GCC, I was grateful for the ways students had expanded their worldview and opened themselves up to the possibilities of leading differently. The values of Ubuntu have beautifully grounded this global experience, and the emergence of critical leaders is underway.

### 4.8 **Week Three: Compassion and Empathy**‐**Gender‐Minoritized Voices Who Lead**

I'm storing a few key tools in my leadership toolbox: the importance of cultural humility, the courage to question simplified narratives, and the value of relational leadership where empathy, openness, and trust take center stage. I also began reflecting more deeply on my “STEM story” and how leadership in STEM must center inclusivity, cross‐cultural understanding, and ethical responsibility. ∼N.O.

As the GCC began its final week, we were full of curiosity and hope for what could be. Our last touchpoints this week elevated the Ubuntu values of compassion and empathy; these values remind us to care for others with solidarity and support (Udah et al. 2025). We observed how these values were actualized during our time spent with the dynamic scientists who founded HER Wines, a Black owned, women‐owned winery in Paarl, South Africa. The team at HER Wines shared how they leveraged their navigational and social capitals to successfully traverse the White, male‐dominated wine industry. Students were able to see in practice the benefits of strategic networking and allyship to actualize one's goals.

Not only did the founders of HER Wines curate an award‐winning wine, but they used their financial and social capitals to pay it forward and give back to the community via academic scholarships for women. It was encouraging to see the ways they show solidarity for other women and supported their well‐being and development. Further, they demonstrated how important self‐efficacy (i.e., the belief in oneself to accomplish a task; Rittmayer and Beier 2008) is for leadership practice. Each woman who received a scholarship for university was able to actualize their goals and dreams. Their efficacy increased because of compassion, empathy, and opportunity. Through this vicarious experience (i.e., learning by observing others; Bandura 1997) with HER Wines, GCC student C.O., discovered how her own self‐efficacy had grown. She posited:

The biggest lesson I learned was through the Her Wines experience which cemented the importance of truly believing in yourself and your ability to accomplish things in male dominated fields. Learning about how all the founders and workers got to where they are today was definitely a big reminder that not everything comes easy but if you put your mind to something, it will be worth it to complete it. It also taught me to put value into my relationships with others because the support you receive from those around you can be really motivating and a critical part of your decision making.

Every touchpoint, activity, meal, excursion, speaker, or site visit expanded the perspectives of each student. The week concluded with the voices of the twelve students who shaped and defined this 3‐week GCC experience. Utilizing photo elicitation pedagogy, students collected photographs and artifacts that represented topics and discussions from class sessions, guest lectures, site visits, and excursions. These artifacts illustrated core memories created during the program and reflected key leadership lessons learned for each student. During the last class, students culminated the experience by delivering a presentation of their photographs and artifacts sharing the insights obtained throughout our time in Cape Town.

## 5 Lessons Learned: I Am Because We Are ‐ Ubuntu In Practice

“I'm feeling grateful for the experiences so far and excited for what's to come. This trip is already reshaping how I see myself and the world, and I'm ready to keep learning, unlearning, and growing.” ∼ K.P.

At the conclusion of the GCC, students completed one final assignment, a ‘letter to themselves.’ This letter served as a final reflection and personal call to action. Students were encouraged to consider the insights they had over the 3 weeks in South Africa and reflect on their hopes and dreams for the future as an emerging STEM leader. They were invited to consider how, if at all, this experience has shaped their understanding of leadership, the importance of diverse voices in leadership, and their overall personal, professional, and leadership values and practices. With their leadership capacities and competencies foregrounded, and their social identities centered, participants were ultimately asked: How can they be the STEM leaders we've been waiting for? To ensure students could be their most vulnerable selves, these letters were not read. They will be printed and mailed to each participant once a year has passed from the conclusion of the GCC.

This article could never fully capture every aspect of this global learning experience. However, the leaders who emerged from this experience shared some important lessons learned that I summarize below. I offer these as both a moment of reflection and as a suggested framework through which leadership educators can apply the values of Ubuntu for critical leadership development, within and apart from a study abroad program.

### 5.1 Eight Ways to Lead With Ubuntu Values

1. **Disrupt**: The status quo, the single story, the dominant narrative. Change can only emerge when we make space to learn new things and unlearn things that no longer serve us or the collective.
2. **Center counter‐narratives**: You will never know a different way, until you make space to learn from others. Listening to people's stories, values, and life experiences is the first step in genuinely understanding them as a leader.
3. **Be Present**: Presence—both physical and emotional—is important. Being present means more than just showing up; it means being attentive, adaptable, and aware of your environment and the people around you.
4. **Forge meaningful relationships**: I am because we are, is more than a statement, but an invitation to be in community with others. Leading with purpose, on purpose, and alongside meaningful partnerships is a recipe for success.
5. **Lead with the greater good in mind**: Our innovations as STEM leaders will not occur in a vacuum, they will have direct and indirect implications for others. Ensuring the communities we serve are prioritized in our work is of paramount importance as a leader.
6. **Be open to unlearning**: Simplified narratives can bias interpersonal relationships. Leaders have a responsibility to amplify diverse voices and remain open to unlearning. Telling authentic stories is one of the most powerful tools a leader can carry.
7. **Step outside of comfort**: There is value in pushing yourself outside your comfort zone. Being receptive to new experiences fosters development, comprehension, and connection, qualities essential to any leadership role.
8. **Own your story, your voice, your influence**: Leadership often comes from small, everyday actions—listening, encouraging others, and being confident enough to show up as yourself. Leadership does not always come from having the most resources or the loudest voice, but from standing firm in your values and creating room for others.

### 5.2 Faculty Reflection: *“I am My Ancestor's Wildest Dreams”*

Dreams deferred can come true. I successfully led my first study abroad course, enveloped in the ancestral air, soil, and energy of my fore parents of the African Diaspora. Representation Matters! It is not lost on me the incredible gift and responsibility I shepherded to bring this experience to fruition. I had the esteem privilege of making space for students who have never gone abroad. I observed how students’ efficacy deepened as their social identities were appreciated and celebrated in a new cultural context. Alongside my students, I navigated the discomfort of unlearning information that biased our perspectives, reiterating through each interaction why diverse voices in leadership and STEM matter. Each moment was a powerful lesson and positively shaped my study abroad experience as a faculty member.

This experience reiterated how important study abroad opportunities are for minoritized voices in higher education, especially in STEM. It is equally crucial to go against the grain, to disrupt. Although I am sure GCC programs in other countries have been successful, South Africa's unique history coupled with the intersectional voices “of people who are subordinated within domestic and global expressions of racism, sexism, capitalism, colonialism, and similar systems of political domination and economic exploitation” (Hill Collins 2019, p. 10) cannot be duplicated in other places. I am grateful to have had the opportunity to work with amazing colleagues and learn alongside wonderful students during this experience.

I accept the challenge to recreate this dynamic learning experience for the next cohort of STEM leaders. Although slight adjustments may be offered, from assignments to excursions/site visits, the essence of the Leadership and Technology GCC will remain steadfast. The values of Ubuntu inspire me. Guided by the wisdom and learning achieved by the inaugural GCC cohort, I am motivated to support the next generation of global STEM leaders. I am because THEY are!
